# Cost-Effectiveness of Laparoscopic Sleeve Gastrectomy for Chinese Patients

**DOI:** 10.1007/s11695-024-07330-1

**Published:** 2024-07-09

**Authors:** Weihua Yu, Jionghuang Chen, Luqi Fan, Chenli Yan, Linghua Zhu

**Affiliations:** 1 Department of General Surgery, Sir Run Run Shaw Hospital, School of Medicine, Zhejiang University, Hangzhou, China; 2 School of Business Administration, Capital University of Economics Business, Beijing, China

**Keywords:** Cost-effectiveness, Quality adjusted life years (QALY), Body mass index (BMI), Obesity, Bariatric surgery, Laparoscopic sleeve gastrectomy (LSG)

## Abstract

**Background:**

Laparoscopic sleeve gastrectomy (LSG) is the most popular bariatric surgery procedure in China. However, its cost-effectiveness in Chinese patients is currently unknown.

**Objectives:**

This study aims to assess the cost-effectiveness of LSG vs no surgery in Chinese patients with severe and complex obesity, taking into account both healthcare expenses and the potential improvement in health-related quality of life (HRQoL).

**Methods:**

A retrospective cohort study was conducted, encompassing 135 Chinese patients who underwent LSG between January 3, 2022 and December 29, 2022, at a major bariatric center. The study evaluated the cost-effectiveness from a healthcare service perspective, employing the incremental cost-effectiveness ratio (ICER) for quality-adjusted life years (QALYs) gained. The analyses compared LSG with the alternative of not undergoing surgery over a 1-year period, using actual data, and extended to a lifetime horizon by projecting costs and utilities at an annual discount rate of 3.0%. Subgroup analyses were undertaken to explore cost-effectiveness variations across different sex, age and BMI categories, and diabetes status, employing a one-way analysis of variance (ANOVA). To ensure the reliability of the findings, one-way and probabilistic sensitivity analyses were executed.

**Results:**

The results indicated that 1-year post-LSG, patients achieved an average total weight loss (TWL) of (32.7 ± 7.3)% and an excess weight loss (EWL) of (97.8 ± 23.1)%. The ICER for LSG compared to no surgery over a lifetime was $4,327/QALY, significantly below the willingness-to-pay (WTP) threshold for Chinese patients with severe and complex obesity. From a lifetime perspective, LSG proved to be cost-effective for all sex and age groups, across all BMI categories, and for both patients with and without diabetes. Notably, it was more cost-effective for younger patients, patients with higher BMI, and patients with diabetes.

**Conclusions:**

LSG is a highly cost-effective intervention for managing obesity in Chinese patients, delivering substantial benefits in terms of HRQoL improvement at a low cost. Its cost-effectiveness is particularly pronounced among younger individuals, those with higher BMI, and patients with diabetes.

**Graphical Abstract:**

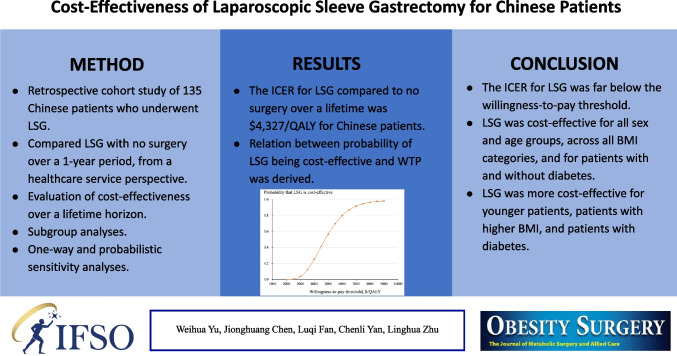

## Introduction

Obesity has become a global epidemic and been identified as a risk factor for numerous diseases such as type 2 diabetes mellitus (T2DM), hypertension, cardiovascular diseases, and certain forms of cancer [1, 2]. In China, the rapid economic development, urbanization, and lifestyle changes over the past several decades have led to a dramatic rise in the prevalence of obesity, which affects about one-sixth of adults ($\ge$ 18 years) and poses a substantial public health challenge and economic burden [3]. Among Chinese patients of severe and complex obesity, bariatric surgery has emerged as an increasingly popular intervention for achieving significant and sustained weight loss and improving obesity-associated medical problems, with more than 25,000 procedures performed in 2022 alone [4]. Among the various bariatric procedures, laparoscopic sleeve gastrectomy (LSG) has become the dominant form of surgery in China, accounting for nearly 85% of all bariatric operations conducted in 2021–2022 [5]. LSG involves the removal of a large portion of the stomach to limit food intake without altering the intestines, and is recognized for its simplicity and safety [6].

The rapid escalation of obesity rates in China underscores an urgent need to evaluate the economic outcomes of bariatric interventions, given the procedure’s rising prevalence and its potential to mitigate the extensive public health and economic impacts of obesity. Laparoscopic Roux-en-Y gastric bypass (LRYGB), a procedure accounting for around 3% of bariatric surgeries in China [5], has been shown to be cost-effective for Chinese patients with T2DM over a 4-year horizon compared to conventional management [7]. However, the cost-effectiveness of LSG, the dominant bariatric surgery procedure among Chinese patients, remains unclear.

This study aims to evaluate the cost-effectiveness of LSG for Chinese patients with obesity by analyzing its impact on weight loss, improvement in quality of life, and healthcare costs. A substantial body of papers has examined the cost-effectiveness of LSG in various countries [8–12]. However, the majority of these investigations have relied on simulated patient cohorts with parameters extracted from multiple sources. Contrasting with simulation studies, we employ empirical data extracted from medical records. To our knowledge, this is the first study that establishes the cost-effectiveness of LSG in China.

## Methods

### Study Design and Population

This study employed a retrospective cohort design to assess the cost-effectiveness of LSG in Chinese patients with severe and complex obesity. From January 3, 2022, through December 29, 2022, 549 LSG procedures were performed at the bariatric center of a tertiary hospital. Patients were eligible for bariatric surgery if they had a body mass index (BMI) ≥ 32.5 kg/m^2^ or BMI ≥ 27.5 kg/m^2^ with at least two obesity-related medical conditions, such as T2DM, hypertension, or dyslipidemia, following the Chinese bariatric surgery guidelines. All surgeries were performed by the same surgeon team according to the standard procedure [13]. Among the patients, 154 individuals (28.1%) completed the 1-year follow-up. Furthermore, we excluded patients lacking complete medical records on critical items (e.g., age, cost, BMI), resulting in a final sample of 135 patients. For each patient, we could extract demographic and perioperative clinical data (e.g., weight, BMI, blood pressure, co-occurring health conditions) and data collected at follow-up visits approximately 12 months after the surgery. We evaluated the cost-effectiveness of the LSG procedure against the scenario of no surgery over the 1-year time horizon using empirical data and the lifetime horizon by projecting costs and utilities throughout lifetime. In doing so, the study population was matched to a control group of the same individuals had they not undergone LSG.

### Costs and Utilities

The cost-effectiveness analysis was conducted from the healthcare system perspective. Cost data of LSG were extracted from electronic medical records, covering costs of the surgery, hospitalization, consultation, medical tests, medications and follow-up visits, and excluding any healthcare expenditures linked to the treatment of health issues associated with obesity. The original cost data were reported in CNY and were converted to US dollars according to the average exchange rate in 2022 (1 USD = 6.73 CNY) [14].

Health utilities were measured by HRQoL and QALY. HRQoL provided the quality weights (utility values) that represented the preference for a given health state relative to perfect health (scored as 1) or death (scored as 0). These utility values were then used to adjust the life years gained from a medical intervention, resulting in QALYs. To derive the post-surgery QALYs, we applied a HRQoL improvement of 0.0055 QALYs per BMI unit decrease (i.e., BMI-HRQoL coefficient) based on the EQ-5D utility scores in published research on Chinese population [15]. We noted that the magnitude of the improvement rate was close to that of the US population [8]. We obtained baseline annual medical cost per capita ($223) from the China Health Statistics Yearbook 2022 published by the National Health Commission of China. We assumed this annual cost grew at a rate of 2.9% to account for the effect of aging [16].We performed analyses separately for time horizon of 1 year post surgery to align with the retrospective evidence and for the remaining lifetime of the patients by projecting long-term costs and utilities.

For lifetime analysis, we estimated the total costs and total utilities of the two scenarios (LSG and no surgery) over the lifetime horizon. Given the heterogeneity of the patients undergoing LSG, assuming a uniform lifetime span for all patients would be inappropriate. Therefore, we estimated the life expectancy of each participant adjusted by age and sex based on the life tables for China released by the World Health Organization [17]. We assumed the postoperative costs and health benefits (health states) would decline at a rate of 3% per annum, a discounting rate commonly used in prior cost-effectiveness analysis [9].

Cost and utility outcomes were combined into ICER, defined as the difference in costs between two possible interventions (LSG vs no surgery), divided by the difference in their effectiveness. The ICER was compared to the willingness-to-pay (WTP) threshold for China to determine the cost-effectiveness of LSG. We used the standard WTP threshold ($38,160 per QALY) as 3 times the GDP per capita of China, as well as a more conservative estimate of WTP threshold of $19,019 per QALY as reported in literature, equivalent to 1.76 times the GDP per capita [18].

### Statistical Analysis

Analyses were performed from January 15, 2024, to March 31, 2024. Descriptive statistics were used to summarize baseline characteristics. ICERs between subgroups were compared using 1-way analysis of variance (ANOVA) with statistical significance estimated at p < 0.05 in 2-sided tests. Statistical analyses were performed using R version 4.2.3 (R Project for Statistical Computing).

### Subgroup and Sensitivity Analysis

To examine the uncertainty of outcomes caused by the subpopulations, we conducted exploratory subgroup analyses, including: patients with diabetes vs patients without diabetes, and across different sex, age and BMI groups shown in Table 1. To investigate the robustness of the baseline results, we performed 1-way sensitivity analyses to explore the impact of variations in intervention costs. Across different intervention costs, ICER could be derived and the value at which the ICER crossed the WTP threshold was determined. Probabilistic sensitivity analyses were conducted to evaluate the effect of simultaneously varying multiple parameters, including intervention costs, discount rate, baseline annual medical costs, and health utilities. Intervention costs were varied according to a Gamma distribution. Uniform distributions were assumed for baseline annual medical cost per capita, discount rate and BMI-HRQoL coefficient, all of which could vary 25% from the baseline value. A Monte-Carlo simulation of 10,000 iterations based on prespecified parameter distributions was performed to determine the likelihood that LSG is cost effective across different WTP thresholds.

**Table 1:** Baseline characteristics of patients in the study population

**Characteristic**	**Overall** (N = 135)	**Women** (N = 111)	**Men** (N = 24)
Age, years, mean ± SD^†^	32.5 ± 8.0	32.2 ± 8.0	33.7 ± 8.1
Age group, N (%)			
18 to 29 years	53 (39.3)	46 (41.4)	7 (29.2)
30 to 35 years	40 (29.6)	30 (27.0)	10 (41.7)
≥ 36 years	42 (31.1)	35 (31.5)	7 (29.2)
Weight, kg, mean ± SD	101.3 ± 15.3	97.2 ± 11.8	120.1 ± 16.0
BMI, kg/m^2^, mean ± SD	36.9 ± 3.8	36.4 ± 3.6	39.1 ± 3.9
BMI group, N (%)			
30 to < 35 kg/m^2^	45 (33.3)	40 (36.0)	5 (20.8)
35 to < 40 kg/m^2^	64 (47.4)	55 (49.5)	9 (37.5)
≥ 40 kg/m^2^	26 (19.3)	16 (14.4)	10 (41.7)
Hypertension, N (%)	62 (45.9)	46 (41.4)	16 (66.7)
Systolic, mmHg, mean ± SD	131.7 ± 22.9	129.5 ± 23.0	141.7 ± 19.6
Diastolic, mmHg, mean ± SD	84.5 ± 16.8	84.9 ± 14.0	82.7 ± 26.6
Diabetes, N (%)	35 (25.9)	28 (25.2)	7 (29.2)
HbA1c, %, mean ± SD	6.1 ± 1.2	6.1 ± 1.3	6.1 ± 0.8
Fatty liver disease, N (%)	127 (94.1)	103 (92.8)	24 (100.0)
Obstructive sleep apnea, N (%)	44 (32.6)	35 (31.5)	9 (37.5)
Cost, USD, mean ± SD	6,501 ± 297	6498 ± 302	6,513 ± 278

^†^SD: standard deviation

## Results

### Demographic and Clinical Characteristics

Table 1 showed the baseline characteristics of the patients by sex. The overall patient cohort (n = 135) included 82% (111) women and 18% (24) men, with a mean ($$\pm$$ SD) age at LSG of 32.5 $$\pm$$ 8.0 years, an average weight of 97.2 $$\pm$$ 11.8 kg and an average BMI of 36.4 $$\pm$$ 3.6 kg/m^2^. For female patients, the presurgical. At the 1-year follow-up after the surgery, the mean weight of female patients decreased to 65.2 kg and the mean BMI dropped to 24.4 kg/m^2^, corresponding to a 33% drop in weight and BMI. Before undergoing LSG, the male patients had a mean weight of 120.1 kg and a mean BMI of 39.1 kg/m^2^. The postsurgical weight and BMI at the 1-year follow-up were 80.1 kg and 26.1 kg/m^2^, indicating a 33% drop in both weight and BMI. The mean cost of all patients was $6,501 $$\pm$$ 8.0. The difference between male and female patients in age, weight loss (%), BMI decrease (%) and cost was insignificant.

### Weight Loss, HRQoL and QALYs Gained

Table 2 compared weight change, cost, HRQoL, and cost-effectiveness for LSG and the case of no surgery. Postoperative weight loss was expressed by percentage of total weight loss (TWL), percentage of excess weight loss (EWL) and BMI loss. To derive EWL, we assumed an ideal BMI of 24 kg/m^2^, which corresponded to the diagnostic threshold of overweight in China. Pre- and postoperative characteristics, costs and utilities were compared (Table 2). One year after undergoing LSG, the study population had an average weight of $67.8\pm 10.9$ kg and an average BMI of $24.7\pm 2.9$ kg/m^2^, achieving $\left(32.7\pm 7.3\right)\%$ TWL, $\left(97.8\pm 23.1\right)\%$ EWL, and 12.2 $\pm$ 3.3 kg/m^2^ BMI reduction.

**Table 2:** Weight loss and HRQOL of the study population

	LSG	No bariatric surgery
TWL, %, mean ± SD	32.7 ± 7.3	
EWL, %, mean ± SD	97.8 ± 23.1	
BMI loss, kg/m^2^, mean ± SD	12.2 ± 3.3	
1-year time horizon		
QALY	0.94	0.87
∆QALY	0.07	
Cost, $	6,401	223
∆Cost, $	6,178	
ICER, $/QALY	88,258	
Lifetime time horizon		
QALYs	15.98	14.63
∆QALY	1.35	
Cost, $	17,130	11,288
∆Cost, $	5,842	
ICER, $/QALY	4,327	

The average 1st-year cost amounted to $6,401 per individual in the scenario involving LSG for all, and $223 in the case of no surgery. Patients undergoing surgery would, on average, gain 0.07 QALYs in the 1st-year. ICER, the cost per QALY gained, was estimated at approximately $88,258 over the 1-year span, and exceeded the WTP threshold by a large margin. For the lifetime horizon, LSG led to a gain of 1.35 QALYs at a cost of $5,842 and an ICER of $4,327 per QALY, which was smaller than the WTP threshold.

### Subgroup Analysis

Table 3 reported subgroup analysis by sex, age, BMI and diabetes status. The results revealed that the lower ICER was achieved for the younger patients, patients with higher BMI, and patients with diabetes. The difference between male and female patients was insignificant.

**Table 3:** Subgroup analysis by sex, age, BMI and diabetes status

Group	Scenario	QALYs	∆QALY	Cost ($)	∆Cost ($)	ICER ($/QALY)	P-value†
By Sex, N (%)							0.867
Female, 111 (82)	LSG	16.25	1.35	17,181	5,839	4,325	
	No surgery	14.90		11,341			
Male, 24 (18)	LSG	14.76	1.34	15,704	5,854	4,357	
	No surgery	13.42		9,850			
By Age, N (%)							< 0.001
18 to 29 years, 53 (39)	LSG	17.36	1.47	18,360	5,741	3,893	
	No surgery	15.89		12,618			
30 to 35 years, 40 (30)	LSG	15.91	1.35	16,835	5,915	4,373	
	No surgery	14.56		10,920			
≥ 36 years, 42 (31)	LSG	14.31	1.19	15,179	5,900	4,969	
	No surgery	13.12		9,279			
By BMI, N (%)							< 0.001
30 to < 35 kg/m^2^, 45 (33)	LSG	16.11	1.13	16,824	5,752	5,104	
	No surgery	14.98		11,072			
35 to < 40 kg/m^2^, 64 (47)	LSG	16.14	1.41	17,130	5,885	4,161	
	No surgery	14.73		11,245			
≥ 40 kg/m^2^, 26 (19)	LSG	15.37	1.57	16,559	5,891	3,746	
	No surgery	13.80		10,668			
By Diabetes, N (%)							< 0.001
No, 35 (26)	LSG	15.50	1.19	16,458	5,886	4,926	
	No surgery	14.31		10,572			
Yes, 100 (74)	LSG	16.15	1.40	17,079	5,827	4,153	
	No surgery	14.75		11,253			

^†^ P-value was based on 1-way ANOVA for subgroup comparison

### Sensitivity Analysis

**Fig. 1:**
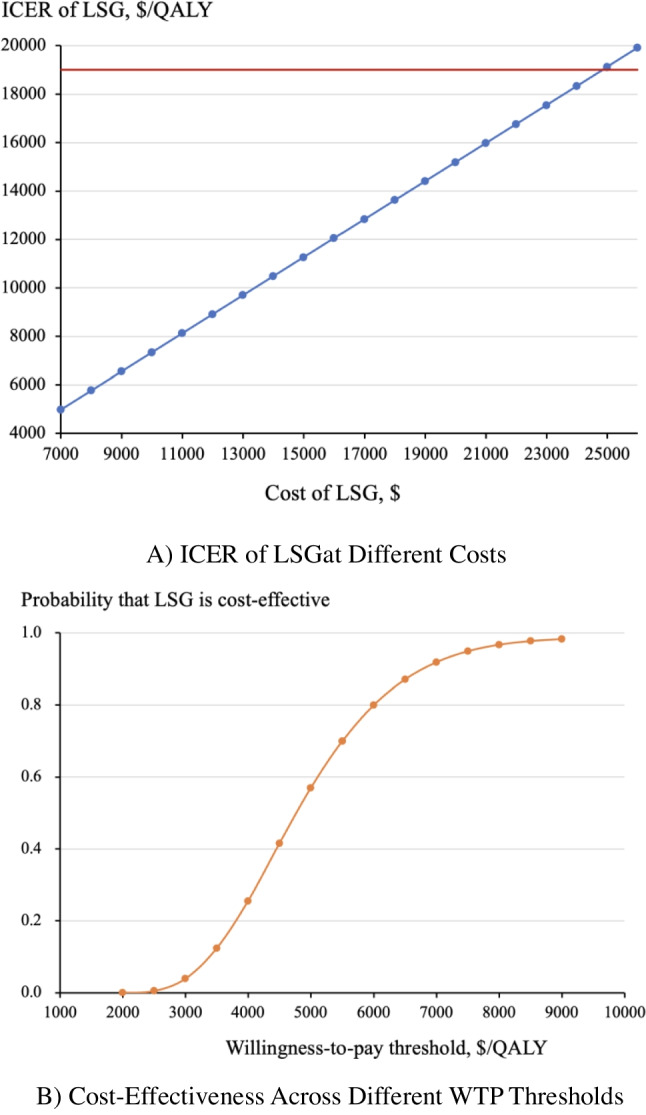
One-Way and Probabilistic Sensitivity Analyses. **A** ICER of LSG at Different Costs. **B** Cost-Effectiveness Across Different WTP Thresholds

One-way sensitivity analyses showed that if the cost of LSG increased to more than $24,541, LSG would no longer be considered cost-effective (Fig. 1A). The cost-effectiveness acceptability curve generated through probabilistic sensitivity analyses revealed that the probability of LSG being cost-effective increased from 57 to 98% when the WTP threshold increased from $5,000/QALY to $9,000/QALY (Fig. 1B). LSG was cost-effective 100% of the time for the baseline WTP threshold.

A. The cost-effectiveness was assessed over the lifetime horizon by varying the cost of LSG while keeping all other parameters constant. The horizontal red line represented the WTP threshold of $19,019 per QALY. LSG remained cost-effective until its cost increased to more than $24,541, almost four times the current cost.

B. The cost-effectiveness was assessed over the lifetime horizon was assessed through the Monte Carlo simulation, which simultaneously varied LSG cost, discount rate, baseline annual medical cost and BMI-HRQoL coefficient. At a willingness-to-pay threshold of $19,019, LSG was cost-effective 100% of the time.

## Discussion

Our analyses address a topical and critical issue of economic assessment for LSG, the dominant bariatric procedure in China. We establish for the first time, to our knowledge, the cost-effectiveness of LSG in Chinese patients, using a mixed, empirical method that utilizes clinical data, patient records and public data sources. The ICER of LSG at $4,327/QALY is particularly cost-effective compared to no surgical intervention, assuming a WTP threshold of $19,019 per QALY. This suggests that, from an economic standpoint, LSG provides significant value for the resources invested, when compared to no surgery for obesity.

This paper’s findings align with a substantial body of research demonstrating that bariatric surgery is a cost-effective intervention for severe and complex obesity over a lifetime horizon [19]. However, the literature has reported a wide range of ICER values, and direct comparisons of cost-effectiveness are because of differences in patient population and the healthcare system within which the surgery is performed.

Prior studies in other countries generally reported that LRYGB was more cost-effective than LSG [12]. In the Chinese context, an early study estimated that LRYGB incurred an ICER of $19,359/QALY over a 4-year period compared to non-surgical treatment [7]. It is difficult to directly compare this ICER with our results due to different time horizons. If we shortened the time horizon in our paper from lifetime to 4-year, we would obtain an ICER of $23,896/QALY. This value is higher than the reported ICER of LRYGB, thereby providing evidence that LRYGB is also likely to be more cost-effective than LSG in China.

Moreover, the evidence indicates a nuanced landscape where the extent of cost-effectiveness is modulated by patient-specific factors such as age, initial BMI and diabetes status. Younger patients and those with a higher BMI before surgery are shown to derive greater long-term health and economic benefits from bariatric procedures. This finding suggests that early intervention in appropriately selected patients could enhance the cost-effectiveness of bariatric surgery, aligning with our conclusions on the differential impact of bariatric surgery based on patient characteristics.

It is noteworthy that the ICER of LSG is likely to decrease further, thereby making LSG even more cost-effective against no surgery for Chinese patients, for several reasons. First, the rolling-out of the national volume-based procurement is expected to reduce the costs of surgeries including LSG [20]. Additionally, previous research has documented a positive trend in surgical results post-bariatric surgery with declining mortality rates [21], a trend that may extend to China. The synergy of declining costs and enhanced surgical results, assuming other variables remain constant, is anticipated to reduce the ICER of LSG.

### Strengths and Limitations

To our knowledge this is the only study to estimate the cost-effectiveness of LSG in Chinese patients with empirical data. The study’s dual time horizon approach, examining both short-term (1-year) and long-term outcomes, provided a nuanced understanding of LSG’s cost-effectiveness. Subgroup analyses by sex, age, BMI groups, and diabetes status allowed for a detailed understanding of which patient groups derived the most benefit from LSG. One-way and probabilistic sensitivity analyses strengthened the study's findings by testing the robustness of the results under various parameter uncertainties.

Our study has several limitations. First, we made simplifying assumptions when projecting costs and utilities over a lifetime. Our model assumed that BMI would stay constant in patients without surgery, thereby not accounting for potential increases in obesity severity over time. We also assumed costs due to obesity-related health conditions remain stable over time. LSG involved long-term costs beyond the immediate expenses related to the surgical procedure. We ignored various components of the long-term costs, including nutritional supplementation and dietary changes. For nutritional supplementation, all patients were advised to take multivitamins (Centrum®, Haleon) twice a day post-surgery, but the purchasing data were unavailable. Not accounting for long-term costs of LSG would overestimate the LSG’s cost-effectiveness. Second, the current analyses did not account for weight regain, the definition and prevalence of which were subject to ongoing debates [22, 23]. Third, as a retrospective study using existing medical records, the study was constrained due to non-standardized data and incomplete follow-up. The sample of patients with follow-up data represented only a portion of all those undergoing the surgery, potentially introducing selection bias into the cost-effectiveness estimates. Finally, the study population did not include patients aged 65 and above, since Chinese patients were generally younger when undergoing bariatric surgery [5]. Future research may examine the cost-effectiveness of LSG for older patients.

## Conclusion

To our knowledge, this study is the first study that evaluates the cost-effectiveness of LSG in Chinese patients. Based on retrospective clinical data of 135 patients, we found that LSG was highly cost-effective compared to no surgery from the lifetime perspective, with its ICER far surpassing the WTP threshold. Moreover, LSG was cost-effective for both male and female, across all age and BMI groups, and for patients with and without diabetes. Notably, LSG demonstrated enhanced cost-effectiveness among younger patients, those with higher BMI and individuals diagnosed with diabetes.

## Data Availability

Raw data of this study are available from the corresponding author Linghua Zhu upon request.
